# Molecular characteristics of new clonal complexes of *Staphylococcus pseudintermedius* from clinically normal dogs

**DOI:** 10.1080/01652176.2017.1400710

**Published:** 2017-12-12

**Authors:** Jae-Ik Han, Haerin Rhim, Cheol-Ho Yang, Hee-Myung Park

**Affiliations:** a Laboratory of Wildlife Medicine/Diseases, College of Veterinary Medicine, Chonbuk National University, Iksan, Republic of Korea; b Department of Veterinary Internal Medicine, College of Veterinary Medicine, Konkuk University, Seoul, Republic of Korea

**Keywords:** Dog, canine, *Staphylococcus pseudintermedius*, genetic lineage, antibiogram

## Abstract

**Background:** High prevalence of methicillin resistance among clinical isolates of *Staphylococcus pseudintermedius* obtained from dogs was reported in Seoul metropolitan area, South Korea. However, no information on genetic lineage and clonal spread is currently available.

**Objective:** The aim is to identify the genetic diversity of methicillin-resistant or -susceptible *S. pseudintermedius* (MRSP and MSSP, respectively) from healthy dogs.

**Animals and methods:** From 119 healthy dogs, 29 isolates consisting of 20 MRSP and 9 MSSP were collected from June 2013 to February 2014. Phenotypic features, antibiogram, multilocus sequence type (MLST), Staphylococcal cassette chromosome *mec* (SCC*mec*) type and *spa* gene type were analyzed.

**Results:** MLST showed 24 sequence types (STs), including 20 new STs that were genetically distinct from the previous STs in other geographic areas. SCC*mec* typing revealed that all isolates had SCC*mec* type V, a predominant type in North America. *spa* gene typing was successful in only 13 isolates (10 MRSP and 3 MSSP) and revealed two known types (t02 and t06), as well as one novel type (t73).

**Conclusion:** Our cumulative data indicate the presence of various populations of *S. pseudintermedius* in clinically normal dogs in Seoul metropolitan area.

## 1. Introduction

Since the significant emergence of methicillin-resistant *Staphylococcus pseudintermedius* (MRSP), mainly due to clonal spread or horizontal gene transfer acquisitions in veterinary practice (Bannoehr et al. 2007; Black et al. 2009; Perreten et al. 2010; Ruscher et al. 2010), several studies have been conducted to identify geographical patterns of clonal spread and gene transfer/sharing, as well as to identify the potential MRSP founder strains of MRSP clones and their genetic lineages in each geographical area (Moodley et al. 2009; Perreten et al. 2010; Solyman et al. 2013). In particular, multilocus sequence typing (MLST) has revealed the broad geographic dissemination of two major clones: ST71 (Europe, Hong Kong, and Japan), and ST68 (North America). In South Korea, previous studies have revealed a high prevalence of methicillin resistance among clinical isolates of *S. pseudintermedius* obtained from dogs with pyoderma or otitis (Yoo et al. 2010; Yoon et al. 2010). However, no information on genetic lineage, clonal spread, or resistant gene transfer/sharing is currently available for strains from healthy dogs or dogs with MRSP infection from this region.

To identify *S. pseudintermedius* genetic lineages, this study was designed to determine the prevalence of methicillin-susceptible (MS) and MRSP, their antimicrobial resistance profiles, and the genetic diversity among isolates from healthy dogs.

## 2. Materials and methods

### 2.1. Sample collection

From June 2013 to February 2014, 119 nasal swab samples were collected from 119 healthy dogs referred to participating veterinary hospitals in five boroughs (Gwangjin, Songpa, Jungnang, Gangdong, and Seocho) of Seoul and Suwon, South Korea. The procedures and sample handling in this study were approved by the Institutional Animal Care and Use Committee (IACUC; approval number KU13087; date of approval 1 June 2013) of Konkuk University. The nasal swab samples were collected using the Transystem Culture swab transport system (Copan, Brescia, Italy). A sterile wet swab was inserted into the nares and gently rotated to make contact with the nasal septum. After collection, each sample was immediately transferred to the clinical laboratory and cultured for isolation of staphylococci. The following animal information was obtained from the owners: sex, age, and breed of the dog, and medication history (Han et al. 2016). As the study focused on carriage rather than infection, seriously ill dogs, and those with obvious infections were excluded.

### 2.2. Identification of *S. pseudintermedius*

The swab samples were inoculated onto trypticase soy agar plates containing 5% sheep blood and the plates were incubated at 37 °C for 24–48 h. The phenotypic and biochemical identification of *Staphylococcus* spp. was performed as described previously (Yoon et al. 2010; Han et al. 2016). *S. aureus* strain ATCC 25923 (American Type Culture Collection [ATCC], Manassas, VA, USA), *S. pseudintermedius* strain ATCC 49051, and a clinical isolate of *S. epidermidis*, confirmed by species-specific PCR (Martineau et al. 2000) and sequencing, were used as the positive and negative controls, respectively, for the phenotypic and biochemical analyses.

The isolated staphylococci were further identified by 16S ribosomal RNA (16S rDNA) and heat shock protein 60 (*hsp*60) gene sequence analyses (Lane et al. 1985; Hill et al. 2006). Homology between the deduced nucleotide sequences and known *Staphylococcus* spp. was analysed with the BLAST search program (National Center for Biotechnology Information [NCBI], USA) based on Clinical and Laboratory Standards Institute (CLSI) interpretive criteria (CLSI 2008). Finally, species identification for the isolates was confirmed by a multiplex PCR method, as described previously (Sasaki et al. 2010).

### 2.3. Determination of methicillin resistance

Methicillin resistance of isolated *S. pseudintermedius* was identified in two ways: a Kirby–Bauer disc diffusion test with an oxacillin disc (1 μg; Oxoid, Hampshire, UK), and a PCR assay targeting the *mec*A gene, as described previously (Zhang et al. 2005). Quality control of the disc diffusion test was performed using MR (ATCC BAA-44) and MS (ATCC 6538) strains of *S. aureus*. In the oxacillin disc diffusion test, a zone diameter ≤17 mm indicated a resistant strain (CLSI 2013).

When the results from the oxacillin disc diffusion test and the PCR assay detecting the *mec*A gene were discordant (i.e. a sample that yielded a *mec*A PCR amplicon but had a zone diameter ≥18 mm), a PBP2a latex agglutination test (MRSA-Screen; Denka Seiken, Tokyo, Japan) was additionally performed according to the manufacturer's protocol. If the isolate was found to be MR according to both the PCR and the PBP2a latex agglutination test, the isolate was considered a resistant strain.

### 2.4. Minimal inhibitory concentration (MIC)

MICs of 21 antimicrobial agents or conventional antimicrobial combinations (clindamycin, tetracycline, penicillin, chloramphenicol, kanamycin, quinupristin/dalfopristin, vancomycin, gentamicin, trimethoprim, erythromycin, ciprofloxacin, sulfamethoxazole, amoxicillin/clavulanate, trimethoprim/sulfamethoxazole, cefpodoxime, oxacillin, ticarcillin/clavulanate, doxycycline, cefazoline, amikacin, and imipenem) against all isolates were determined according to a broth microdilution method with EUST and COMPAN1F format Sensititre plates (TREK Diagnostic Systems, Cleveland, OH, USA) according to the CLSI guidelines (CLSI 2007, 2014). Quality control of the MIC test was performed using a strain of MS *S. aureus* (ATCC 29 213).

### 2.5. Multilocus sequence typing (MLST) and eBURST analysis

The genetic diversity of the MRSP isolates was determined by MLST of seven genes (*tuf*, *cpn60*, *pta*, *purA*, *fdh*, *sar*, and *ack*), as described previously (Solyman et al. 2013). The deduced sequence of each target gene was compared with known sequences from the PubMLST database (http://pubmlst.org/). The aggregated alleles identified from an isolate were used to retrieve its previously designated sequence type (ST) from the database. An isolate with a novel combination of alleles was assigned a new ST number by the database curator, Vincent Perreten (vincent.perreten@vetsuisse.unibe.ch). Finally, the STs of the MRSP isolates were grouped using eBURST V3 and examined for associations with existing 109 STs previously reported in the MLST database.

### 2.6. SCCmec typing

The SCC*mec* types of the MRSP isolates were determined according to a multiplex PCR-based method, as described previously (Zhang et al. 2005). Briefly, 100–200 ng of extracted bacterial genomic DNA was amplified using nine pairs of primers specific for subtypes I, II, III, IVa, IVb, IVc, IVd, and V, and a primer pair for the *mec*A gene as an internal control of the reaction; strains ATCC BAA-44 (type I), BAA-41 (type II), 33592 (type III), BAA-1683 (type IV), and BAA-2094 (type V) served as PCR positive controls. *Staphylococcus* isolates showing the specific band for the internal control but lacking a SCC*mec* type-specific band were categorized as unclassified with non-typed.

### 2.7. *spa* typing

For *spa* typing, the tandem repeat sequence of the *spa* gene was amplified and sequenced according to previously published protocols (Harmsen et al. 2003; Moodley et al. 2009; Perreten et al. 2010; Ruscher et al. 2010). Additional primer pairs (spaSP-F1: 5′-AATGACAGCCAAGCAAAACC and spaSP-R1: TTTCACCAGGTTGAACGACA; spaSP-F2: 5′-CAGCCAAGCAAAACCTGATT and spaSP-R2: GCATCTTTCGCTTTGTCCAT) were used to type isolates that could not be typed with these published protocols. The tandem repeat pattern of the deduced sequence was classified using its *spa* repeat code and the *spa* type was determined by the arrangement of *spa* repeat codes (Moodley et al. 2009). For novel combinations of *spa* repeat codes, new *spa* types were assigned by the curator Arshnee Moodley (asm@sund.ku.dk).

### 2.8. Statistical analyses

The association between resistance to methicillin and resistance to each of the other antibiotics investigated was determined by using a multivariate logistic regression model. The probability of detecting concurrent antibiotic resistance was also analyzed in the same manner. The final model was built by stepwise selection using Firth's penalized likelihood method due to quasi-complete separation of the data. Odds ratios (ORs) with 95% confidence intervals were calculated to assess the likelihood of association. All statistical analyses were conducted by using SPSS v.22 (IBM, Armonk, NY, USA). For all analyses, a value of *p* < 0.05 was considered significant.

## 3. Results

### 3.1. Identification of *S. pseudintermedius*

From 119 swab samples, *S. pseudintermedius* was isolated from 29 samples (24.4%). Of the 29 isolates, 20 were MR while 9 were MS. Two of the MR showed an inhibition zone diameter larger than 17 mm (18 and 21 mm, respectively) for the oxacillin disc. However, both isolates were considered MRSP, based on positive results of both the *mec*A PCR and the PBP2a latex agglutination test.

### 3.2. MIC

While all MRSP isolates displayed resistance to more than one antimicrobial agent, five of the nine MSSP isolates showed multiple resistance. In both the MRSP and MSSP isolates, resistance to 6–10 antimicrobial agents was the most commonly seen, and 60% of MRSP isolates showed resistance to more than 11 antimicrobial agents. While all MRSP and MSSP isolates were susceptible to amikacin and imipenem, both groups were commonly resistant to sulfamethoxazole (86.2%), penicillin (86.2%), kanamycin (79.3%), tetracycline (72.4%), and trimethoprim (72.4%). While resistance to sulfamethoxazole was the most common antimicrobial resistance found in MRSP isolates (100%), resistance to penicillin was the most common in MSSP isolates (66.7%). The resistance profiles of each isolated strain are summarised in Table 1.

**Table 1.** Molecular characteristics of the 29 *Staphylococcus pseudintermedius* strains isolated from healthy dogs.

					Antimicrobial resistance profile^e^																		
MR^a^ status	Strain	MLST^b^	*Spa*	SCC*mec*	CLI	TE	P	CHL	KAN	SYN	VAN	GEN	TMP	E	CIP	SMX	AUG	SXT	POD	OXA	TIM	DOX	FAZ
MR^a^	7	361	N^c^	V		R	R		R			R	R	R	R	R	R	R	R	R	R	R	R
	26	363	N	V			R		R				R			R				R			
	31	363	N	V		R	R		R							R				R		R	
	29	364	N	N	R^f^	R	R		R			R	R	R		R	R	R	R	R	R	R	R
	34	365	t02	V		R	R		R			R	R			R	R	R	R	R	R	R	
	70	365	t02	V		R	R		R				R		R	R		R	R	R	R	R	R
	78	365	t02	V		R	R		R			R	R			R		R	R	R	R	R	
	157	365	t02	V		R	R		R			R	R		R	R	R	R	R	R	R	R	R
	175	365	t02	V		R	R		R			R	R			R		R	R	R	R	R	
	46	366	N	V		R	R		R			R	R		R	R				R		R	
	48	367	t06	V			R		R			R			R	R			R	R			
	50	368	N	V												R				R			
	76	370	N	V	R	R	R		R			R	R	R	R	R		R		R		R	
	133	371	t06	V	R	R	R		R				R	R	R	R		R		R			
	101	372	t06	V		R	R		R			R	R		R	R			R	R		R	
	152	373	N	V	R	R	R	R		R	R		R	R		R			R	R			
	86	54	N	V		R	R		R			R	R		R	R				R			
	66	112	t06	N	R	R	R		R			R	R	R		R	R	R	R	R	R	R	R
	103	121	t06	V	R	R	R	R	R				R	R	R	R		R		R			
	149	309	N	V	R	R	R	R	R			R	R	R	R	R				R			
MS^a^	15	373	N	NT^d^												R							
	38	374	N	NT			R																
	40	375	N	NT																			
	44	376	N	NT	R	R	R	R	R			R	R	R	R							R	
	60	377	t73	NT	R	R	R	R	R			R	R	R	R	R							
	62	378	t06	NT	R	R	R		R			R	R	R		R		R				R	
	63	379	t73	NT	R	R	R		R			R	R	R		R		R				R	
	84	380	N	NT	R		R	R	R			R		R		R							
	104	381	N	NT														R					
				Resistance breakpoint for antimicrobials^g^	≥4	≥16	≥0.25	≥32	≥64	≥4	≥16	≥16	≥16	≥8	≥4	≥512	>8/4	≥4/76	≥8	≥4	≥16/2	≥16	≥32

^a^MR, methicillin-resistance; MS, methicillin-susceptible.

^b^MLST, multilocus sequence type.

^c^N, no amplification in PCR.

^d^NT, not tested.

^e^CLI, clindamycin; TE, tetracycline; P, penicillin; CHL, chloramphenicol; KAN, kanamycin; SYN, Quinupristin/dalfopristin; VAN, vancomycin; GEN, gentamicin; TMP, trimethoprim; E, erythromycin; CIP, ciprofloxacin; SMX, sulfamethoxazole; AUG, amoxicillin/clavulanate; SXT, trimethoprim/sulfamethoxazole; POD, cefpodoxime; OXA, oxacillin; TIM, ticarcillin/clavulanate; DOX, doxycycline; FAZ, cefazoline.

^f^R, resistant.

^g^References, CLSI M100-S17 (for AUG, POD, TIM and FAZ) and M100-S24 (for all the other antimicrobial agents).

A statistically significant correlation was detected among 13 of the 21 antibiotics in the incidence of resistance to them (Table 2). The highest incidence of concurrent antibiotic resistance in the isolates was for tetracycline and trimethoprim, an association that was statistically significant (*p* = 0.001; OR = 140.0). The presence of oxacillin resistance was significantly correlated with the incidence of tetracycline or trimethoprim resistance (*p* = 0.033; OR = 7.1 for both antibiotics). The resistance to clindamycin, chloramphenicol, quinupristin/dalfopristin, vancomycin, ciprofloxacin, ticarcillin/clavulanate, amikacin, or imipenem was not found to correlate with resistance to other antibiotics.

**Table 2.** Concurrent detection of antibiotic resistance in *S. pseudintermedius* isolates and the strength of the association.

Reference antibiotics	Associated antibiotics	*p* Value	Odds ratio	95% CI
Oxacillin	Tetracycline	0.033	7.1	1.2–42.8
	Trimethoprim	0.033	7.1	1.2–42.8
Tetracycline	Oxacillin	0.033	7.1	1.2–42.8
	Kanamycin	0.008	15.8	2.1–122.1
	Gentamicin	0.019	9.6	1.5–63.5
	Trimethoprim	0.001	140.0	7.7–2550.4
	Erythromycin	0.036	11.4	1.2–110.4
	Sulfamethoxazole	0.048	12.0	1.0–141.3
	Trimethoprim/Sulfamethoxazole	0.036	11.4	1.2–110.4
Kanamycin	Tetracycline	0.008	15.8	2.1–122.1
	Gentamicin	0.012	20.4	2.0–211.8
	Trimethoprim	0.008	15.8	2.1–122.1
	Sulfamethoxazole	0.031	15.8	1.3–192.5
	Doxycycline	0.044	10.5	1.1–103.5
Gentamicin	Tetracycline	0.019	9.6	1.5–63.5
	Kanamycin	0.012	20.4	2.0–211.8
	Trimethoprim	0.019	9.6	1.5–63.5
	Doxycycline	0.009	11.7	1.8–74.2
Trimethoprim	Oxacillin	0.033	7.1	1.2–42.8
	Tetracycline	0.001	140.0	7.7–2550.4
	Kanamycin	0.008	15.8	2.1–122.1
	Gentamicin	0.019	9.6	1.5–63.5
	Erythromycin	0.036	11.4	1.2–110.4
	Sulfamethoxazole	0.048	12.0	1.0–141.3
	Trimethoprim/sulfamethoxazole	0.036	11.4	1.2–110.4
	Doxycycline	0.023	14.0	1.4–137.3
Erythromycin	Tetracycline	0.036	11.4	1.2–110.4
	Trimethoprim	0.036	11.4	1.2–110.4
Sulfamethoxazole	Tetracycline	0.048	12.0	1.0–141.3
	Penicillin	0.049	11.5	1.0–131.3
	Kanamycin	0.031	15.8	1.3–192.5
	Trimethoprim	0.048	12.0	1.0–141.3
Penicillin	Sulfamethoxazole	0.049	11.5	1.0–131.3
Amoxicillin/clavulanate	Cefazoline	0.003	92.0	4.7–1790.1
Trimethoprim/sulfamethoxazole	Tetracycline	0.036	11.4	1.2–110.4
	Trimethoprim	0.036	11.4	1.2–110.4
	Cefpodoxime	0.047	5.3	1.0–27.8
	Doxycycline	0.008	10.1	1.8–56.0
Cefpodoxime	Doxycycline	0.018	9.0	1.5–55.5
	Trimethoprime/sulfamethoxazole	0.047	5.3	1.0–27.8
Doxycycline	Kanamycin	0.044	10.5	1.1–103.5
	Gentamicin	0.009	11.7	1.8–74.2
	Trimethoprim	0.023	14.0	1.4–137.3
	Trimethoprim/sulfamethoxazole	0.008	10.1	1.8–56.0
	Cefpodoxime	0.018	9.0	1.5–55.5
Cefazoline	Amoxicillin/clavulanate	0.003	92.0	4.7–1790.1

### 3.3. MLST and eBURST diagram analysis

From MLST analysis of the sequence variation at seven loci, the 20 MRSPs yielded 15 STs, which included 11 new STs. Furthermore, all MSSP isolates yielded new, unique STs (Table 1). ST365, a new type, was the most commonly identified MRSP isolate (*n* = 5). None of the MRSP STs were shared with MSSP isolates, except for ST373.

**Figure 1.**
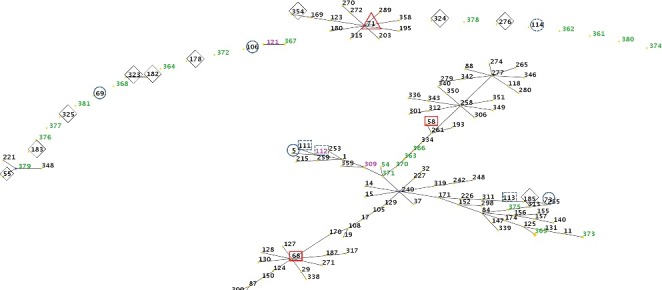
Population snapshot of *Staphylococcus pseudintermedius*. Clusters of related sequence types (STs) from the *S. pseudintermedius* database are displayed as a single eBURST diagram, by setting the group definition to zero of seven shared alleles. Clusters of linked isolates correspond to clonal complexes. The STs identified in the present study are marked in blue (new STs) or purple (previously identified STs) numbers, and previously identified STs are shown with symbols: open blue circle = exclusive to Europe; open red squares = exclusive to North America; open triangles = found in Europe and North America; open blue circles with dotted lines = found in both Europe and Asia; open diamonds = Asia.

In the eBURST diagram, 11 of the 15 MRSP STs were single- or double-locus variants (SVL or DVLs) of the previously identified STs (Figure 1). In particular, none of the MRSP STs showed a genetic association with the strains reported from Asia. Only three of the nine MSSP STs were SLVs of previously identified STs, whereas one of them was a SLV of ST55, which was isolated from Thailand.

### 3.4. Determination of SCC*mec* and *spa* type

SCC*mec* typing was performed on 20 MRSP isolates. The typing of 20 MRSPs revealed only one SCC*mec* type (type V) and two non-typeable cassettes. A segment of the *spa* gene was successfully amplified and sequenced from only 13 isolates (10 MRSPs and 3 MSSPs) by PCR with six sets of *spa* gene-specific primers. Of the 10 *spa*-positive MRSP strains, five strains had *spa* type t02, whereas the remaining had *spa* type t06 (Table 1). For the three MSSP strains, only one was typeable (*spa* type t06) while the other two isolates contained novel repeats sequence (r01r12r12r12r02r02r03r06r05) that were assigned as t73. All isolates assigned to ST365 (*n* = 5) displayed *spa* type t02. The eight remaining *spa* gene-positive isolates showed unique STs. All ST365-t02 isolates possessed the type V SCC*mec* cassette and also shared phenotypically similar antimicrobial resistance profiles (i.e. resistance to tetracycline, penicillin, kanamycin, trimethoprim, sulfamethoxazole, trimethoprim/sulfamethoxazole, cefpodoxime, oxacillin, ticarcillin/clavulanate and doxycycline), whereas other isolates showed various resistance patterns (Table 1).

## 4. Discussion

One of the most interesting findings in this study was that MLST identified the presence of various subtypes, whereas most of the MRSP isolates contained the same SCC*mec* element (type V). MLST did not identify any strains that were genetically identical or closely related to the strains that have been reported as the predominant subtypes in North America (ST68), Europe (ST71), and even northeast Asia (ST71, China and Japan) with the exception of ST55 that was found in Thailand (Bardiau et al. 2013; Perreten et al. 2013). When considering that strains sharing the same genetic background have spread through other geographical areas (Bannoehr et al. 2007; Ruscher et al. 2010), it seems that there has not yet been any broad geographical dissemination of a particular genetic lineage in the metropolitan area of Seoul, Korea.

Our study showed a much higher incidence of methicillin resistance (69.0%) among the isolated *S. pseudintermedius* in healthy dogs than one which had been found in previous reports surveying hospitalized dogs (26.8%) and outpatients with bacterial pyoderma (33.8%) in Korea (Yoo et al. 2010; Yoon et al. 2010). Compared with the prevalence in healthy dogs, the prevalence of MR in this study was also higher than the one reported in other countries (0%–17%; Shimizu et al. 2001; Morris et al. 2006).

In this study, a segment of the *spa* gene was successfully amplified from only 44.8% of the isolates, whereas 16S rDNA and *hsp*60 gene were successfully amplified in all of the isolates. The negative results from the *spa* gene-specific PCRs suggest two possibilities: (1) not all *S. pseudintermedius* have the *spa* gene or its homolog (Perreten et al. 2013) or (2) if the gene or its homolog are present in all *S. pseudintermedius*, it is difficult to detect the sequence by simple PCR because of high sequence diversity. By contrast, the isolates that yielded a *spa* genotype showed only three types, which consisted of two known types (t02 and t06) and a novel type (t73). In particular, t06 strains were of a different MLST subtype, whereas t02 strains were of the same MLST type (ST365). Along with the difficulty in detecting the gene sequence by PCR, our results indicate that *spa* typing has a weaker discriminatory power for distinguishing these bacteria than MLST.

Resistance to14 of 21 antimicrobial agents (66.7%) was significantly correlated with the resistance to other antimicrobial agents. Among them, the associations between tetracycline and trimethoprim (OR = 140.0) was the most significant, reflecting the extensive use of these antimicrobial agents in veterinary practice in Korea. In the case of MRSP isolates, resistance to tetracycline (OR = 7.1) and trimethoprim (OR = 7.1) were both positively correlated with resistance to oxacillin, which is consistent with previous results from European or North American strains (Perreten et al. 2010). However, chloramphenicol resistance was found in only 4 of the 20 isolates that had a different MLST subtype; thus, it was not statistically associated with oxacillin resistance, as predicted by the results from European strains (van Duijkeren et al. 2011).

In conclusion, various genetic lineages of *S. pseudintermedius* are present in healthy dogs in the metropolitan area of Seoul regardless of the presence of MR, although they all belong to a common SCC*mec* type. In particular, MLST showed that 79.2% of the isolates had novel STs that had never been reported previously from other geographical areas. Although this study was conducted with a limited number of strains, our results indicate a unique genetic population of *S. pseudintermedius* in the population of healthy dogs in the metropolitan area of Seoul.

## References

[cit0001] Bannoehr J, Ben Zakour NL, Waller AS, Guardabassi L, Thoday KL, van den Broek AH, Fitzgerald JR 2007 Population genetic structure of the *Staphylococcus intermedius* group: insights into agr diversification and the emergence of methicillin-resistant strains. J Bacteriol. 189:8685–8692. 17905991 10.1128/JB.01150-07 PMC2168937

[cit0002] Bardiau M, Yamazaki K, Ote I, Misawa N, Mainil JG 2013 Characterization of methicilin-resistant *Staphylococcus pseudintermedius* isolated from dogs and cats. Microbiol Immunol. 57:496–501. 23607810 10.1111/1348-0421.12059

[cit0003] Black CC, Solyman SM, Eberlein LC, Bemis DA, Woron AM, Kania SA 2009 Identification of a predominant multilocus sequence type, pulse-field gel electrophoresis cluster, and novel staphylococcal chromosomal cassette in clinical isolates of *mec*A-containing, methicillin-resistant *Staphylococcus pseudintermedius*. Vet Microbiol. 139:333–338. 19604657 10.1016/j.vetmic.2009.06.029

[cit0004] Clinical laboratory standards institute (CLSI) 2007 Performance standards for antimicrobial susceptibility testing; approved standard supplement. Wayne: Clinical and Laboratory Standards Institute CLSI Informational Supplement M100-S17.

[cit0005] Clinical laboratory standards institute (CLSI) 2008 Interpretive criteria for identification of bacteria and fungi by DNA target sequencing; approved guideline. Wayne: Clinical and Laboratory Standards Institute CLSI Informational Supplement MM18-A.

[cit0006] Clinical laboratory standards institute (CLSI) 2013 Performance standards for antimicrobial disk and dilution susceptibility tests for bacteria isolated from animals; approved guideline. 4th ed Wayne: Clinical and Laboratory Standards Institute CLSI Informational Supplement VET01-A4.

[cit0007] Clinical laboratory standards institute (CLSI) 2014 Performance standards for antimicrobial susceptibility testing; approved standard supplement. Wayne: Clinical and Laboratory Standards Institute CLSI Informational Supplement M100-S24.

[cit0008] Han JI, Yang CH, Park HM 2016 Prevalence and risk factors of *Staphylococcus* spp. carriage among dogs and their owners: a cross-sectional study. Vet J. 212:15–21. 27256020 10.1016/j.tvjl.2015.10.059

[cit0009] Harmsen D, Claus H, Witte W, Rothgänger J, Claus H, Turnwald D, Vogel U 2003 Typing of methicillin-resistant *Staphylococcus aureus* in a university hospital setting by using novel software for *spa* repeat determination and database management. J Clin Microbiol. 41:5442–5448. 14662923 10.1128/JCM.41.12.5442-5448.2003 PMC309029

[cit0010] Hill JE, Town JR, Hemmingsen SM 2006 Improved template representation in *cpn*60 polymerase chain reaction (PCR) product libraries generated from complex templates by application of a specific mixture of PCR primers. Environ Microbiol. 8:741–746. 16584485 10.1111/j.1462-2920.2005.00944.x

[cit0011] Lane DJ, Pace B, Olsen GJ, Stahl DA, Sogin ML, Pace NR 1985 Rapid determination of 16S ribosomal RNA sequences for phylogenetic analyses. Proc Nat Acad Sci USA. 82:6955–6959. 2413450 10.1073/pnas.82.20.6955 PMC391288

[cit0012] Martineau F, Picard FJ, Grenier L, Roy PH, Ouellette M, Bergeron MG 2000 Multiplex PCR assays for the detection of clinically relevant antibiotic resistance genes in staphylococci isolated from patients infected after cardiac surgery. The ESPRIT Trial. J Antimicrob Chemother. 46:527–534. 11020248 10.1093/jac/46.4.527

[cit0013] Moodley A, Stegger M, Zakour NLB, Fitzgerald JR, Guardabassi L 2009 Tandem repeat sequence analysis of staphylococcal protein A (*spa*) gene in methicillin-resistant *Staphylococcus pseudintermedius*. Vet Microbiol. 135:320–326. 18995972 10.1016/j.vetmic.2008.09.070

[cit0014] Morris D, Rook K, Shofer F, Rankin SC 2006 Screening of *Staphylococcus aureus*, *Staphylococcus intermedius*, and *Staphylococcus schleiferi* isolates obtained from small companion animals for antimicrobial resistance: a retrospective review of 749 isolates (2003-04). Vet Dermatol. 17:332–337. 16961819 10.1111/j.1365-3164.2006.00536.x

[cit0015] Perreten V, Chanchaithong P, Prapasarakul N, Rossano A, Blum SE, Elad D, Schwendener S 2013 Novel pseudo-staphylococcal cassette chromosome *mec* element (ΨSCC*mec*_57395_) in methicillin-resistant *Staphylococcus pseudintermedius* CC45. Antimicrob Agents Chemother. 57:5509–5515. 23979735 10.1128/AAC.00738-13 PMC3811289

[cit0016] Perreten V, Kadlec K, Schwarz S, Andersson UG, Finn M, Greko C, Moodley A, Kania SA, Frank LA, Bemis DA, et al. 2010 Clonal spread of methicillin-resistant *Staphylococcus pseudintermedius* in Europe and North America: an international multicentre study. J Antimicrob Chemother. 65:1145–1154. 20348087 10.1093/jac/dkq078

[cit0017] Ruscher C, Lubke-Becker A, Semmler T, Wleklinski CG, Paasch A, Soba A, Stamm I, Kopp P, Wieler LH, Walther B 2010 Widespread rapid emergence of a distinct methicillin- and multidrug-resistant *Staphylococcus pseudintermedius* (MRSP) genetic lineage in Europe. Vet Microbiol. 144:340–346. 20181441 10.1016/j.vetmic.2010.01.008

[cit0018] Sasaki T, Tsubakishita S, Tanaka Y, Sakusabe A, Ohtsuka M, Hirotaki S, Kawakami T, Fukata T, Hiramatsu K 2010 Multiplex-PCR method for species identification of coagulase-positive staphylococci. J Clin Microbiol. 48:765–769. 20053855 10.1128/JCM.01232-09 PMC2832457

[cit0019] Shimizu A, Wakita Y, Nagase S, Okabe M, Koji T, Hayashi T, Nagase N, Sasaki A, Kawano J, Yamashita K, et al. 2001 Antimicrobial susceptibility of *Staphylococcus intermedius* isolated from healthy and diseased dog. J Vet Med Sci. 63:357–360. 11307945 10.1292/jvms.63.357

[cit0020] Solyman SM, Black CC, Duim B, Perreten V, van Duijkeren E, Wagenaar JA, Eberlein LC, Sadeghi LN, Videla R, Bemis DA, et al. 2013 Multilocus sequence typing for characterization of *Staphylococcus pseudintermedius*. J Clinl Microbiol. 51:306–310. 10.1128/JCM.02421-12 PMC3536184 23115265

[cit0021] van Duijkeren E, Catry B, Greko C, Moreno MA, Pomba MC, Pyörälä S, Ruzauskas M, Sanders P, Threlfall EJ, Torren-Edo J, et al. 2011 Review on methicillin-resistant *Staphylococcus pseudintermedius*. J Antimicrob Chemother. 66:2705–2714. 21930571 10.1093/jac/dkr367

[cit0022] Yoo JH, Yoon JW, Lee SY, Park HM 2010 High prevalence of fluoroquinolone- and methicillin-resistant *Staphylococcus pseudintermedius* isolates from canine pyoderma and otitis externa in veterinary teaching hospital. J Microbiol Biotechnol. 20:798–802. 20467256

[cit0023] Yoon JW, Lee GJ, Lee SY, Park C, Yoo JH, Park HM 2010 Prevalence of genes for enterotoxins, toxic shock syndrome toxin 1 and exfoliative toxin among clinical isolates of *Staphylococcus pseudintermedius* from canine origin. Vet Dermatol. 21:484–489. 20500497 10.1111/j.1365-3164.2009.00874.x

[cit0024] Zhang K, McClure JA, Elsayed S, Louie T, Conly JM 2005 Novel multiplex PCR assay for characterization and concomitant subtyping of staphylococcal cassette chromosome mec types I to V in methicillin-resistant *Staphylococcus aureus*. J Clinl Microbiol. 43:5026–5033. 10.1128/JCM.43.10.5026-5033.2005 PMC1248471 16207957

